# Input-selective adenosine A_1_ receptor-mediated synaptic depression of excitatory transmission in dorsal striatum

**DOI:** 10.1038/s41598-021-85513-x

**Published:** 2021-03-18

**Authors:** Brandon M. Fritz, Fuqin Yin, Brady K. Atwood

**Affiliations:** 1 Department of Pharmacology and Toxicology, Indiana University School of Medicine, Indianapolis, IN 46202 USA; 2 Department of Pharmacology and Toxicology, Indiana University School of Medicine, 320 W. 15th Street, NB-400C, Indianapolis, IN 46202 USA

**Keywords:** Neuroscience, Neural circuits, Neuronal physiology, Synaptic plasticity

## Abstract

The medial (DMS) and lateral (DLS) dorsal striatum differentially drive goal-directed and habitual/compulsive behaviors, respectively, and are implicated in a variety of neuropsychiatric disorders. These subregions receive distinct inputs from cortical and thalamic regions which uniquely determine dorsal striatal activity and function. Adenosine A_1_ receptors (A1Rs) are prolific within striatum and regulate excitatory glutamate transmission. Thus, A1Rs may have regionally-specific effects on neuroadaptive processes which may ultimately influence striatally-mediated behaviors. The occurrence of A1R-driven plasticity at specific excitatory inputs to dorsal striatum is currently unknown. To better understand how A1Rs may influence these behaviors, we first sought to understand how A1Rs modulate these distinct inputs. We evaluated A1R-mediated inhibition of cortico- and thalamostriatal transmission using in vitro whole-cell, patch clamp slice electrophysiology recordings in medium spiny neurons from both the DLS and DMS of C57BL/6J mice in conjunction with optogenetic approaches. In addition, conditional A1R KO mice lacking A1Rs at specific striatal inputs to DMS and DLS were generated to directly determine the role of these presynaptic A1Rs on the measured electrophysiological responses. Activation of presynaptic A1Rs produced significant and prolonged synaptic depression (A1R-SD) of excitatory transmission in the both the DLS and DMS of male and female animals. Our findings indicate that A1R-SD at corticostriatal and thalamostriatal inputs to DLS can be additive and that A1R-SD in DMS occurs primarily at thalamostriatal inputs. These findings advance the field’s understanding of the functional roles of A1Rs in striatum and implicate their potential contribution to neuropsychiatric diseases.

## Introduction

Adenosine is a natural by-product of cellular respiration processes that accumulates in brain throughout the active, awake circadian cycle^1^. Acting as a neuromodulator, adenosine binds to two functionally opposing G protein coupled receptors in the central nervous system (CNS): G_i/o_-coupled A_1_ receptors (A1Rs) and G_s_-coupled A_2A_ (A2ARs) receptors. A1Rs and A2ARs serve inhibitory and promotional roles, respectively, in neuronal excitability and neurotransmitter release^2,3^. Aberrant adenosine function has been associated with a variety of disorders that substantially impact patients’ quality of life, including substance use^4,5^, movement^6^, and sleep disorders^1,7^. Treatments targeting this understudied system may therefore offer significant therapeutic potential for many diverse ailments. In this regard, the dorsal striatum is a brain region of particular interest given its primary role in the development of adaptive behaviors and its abundant adenosine receptor expression^2,3^.

The dorsal striatum is comprised of functionally heterogeneous medial (DMS) and lateral (DLS) subregions, each receiving unique excitatory glutamatergic inputs driving medium spiny neuron (MSN) activity, the output neurons of the dorsal striatum. These afferents include projections from sensorimotor and prefrontal cortices as well as various thalamic nuclei^8,9^. MSNs regulate basal ganglia output^10^, and ultimately control motor learning^11^, coordination^12^, action selection^13^, and strategic planning of movement^10^. These unique inputs to the dorsal striatal subregions likely explain their specific roles in these complex behaviors. For example, the DMS primarily drives goal-directed behaviors whereas the DLS is critical for the development of habitual behaviors^14,15^. Therefore, alterations in this neurocircuitry have implications for problematic phenotypes associated with various disease states, such as impulsivity and inflexible behavior.

The A1R is by far the most prevalent adenosine receptor within the CNS and is richly expressed in dorsal striatum^16^. Found both pre-^17^ and post-synaptically^18^ within the dorsal striatum, the A1R is coupled to G_i/o_ and its activation has a general inhibitory effect on neural transmission. Depending on its locus, A1R stimulation reduces the likelihood of neurotransmitter release from presynaptic terminals^19^ or hyperpolarizes the postsynaptic cell^20^, thus reducing its excitability. Within the dorsal striatum, application of an A1R agonist acutely reduces glutamate-mediated excitatory postsynaptic potentials (EPSPs) at putative cortical and thalamic inputs^21^, although subregion specificity was not assessed. Trusel et al.^22^ also observed that a 20-min application of the A1R agonist ENBA (200 nM) produced a lingering synaptic depression of glutamate release (30-min post-drug) on to dopamine D1 receptor-expressing direct pathway MSNs within the DLS, indicating a form inhibitory plasticity. However, in this study specific inputs to DLS and DMS were not probed for differential expression of A1R-SD.

Synaptic plasticity within the dorsal striatum is a critical process in neurocircuit dynamics that drives the development of adaptive behaviors^23–25^. For example, disruptions in dorsal striatal synaptic depression have been observed in animal models of inflexible cognitive-behavioral pathologies such as substance use disorders^26^, speech/language disorders^27^, and neurodegenerative diseases^28,29^. Aberrant dorsal striatal A1R-driven synaptic depression may therefore be a critical component of various disorders of behavioral adaptability. Before this can be directly examined, however, A1R-SD must be more thoroughly understood. It is not known whether A1R-SD occurs similarly at cortical or thalamic inputs to DMS and DLS. Given that there are highly distinct sets of afferents that each subregion receives, it is important to define how A1R-SD regulates input to each subregion.

In the current study, we combine brain slice electrophysiology with transgenic mouse technologies to examine sustained effects of pharmacological A1R activation on presynaptic glutamate release specifically from cortical and thalamic inputs to the DMS and DLS. Our findings demonstrate that A1R-SD in the dorsal striatum displays both input- and subregion-specificity and thus has significant potential to alter broader neurocircuit function in a meaningful way.

## Methods

### Animals

Adult C57BL/6J mice (postnatal day 56 ± 3) were ordered from the Jackson Laboratory (Bar Harbor, ME, USA) and allowed at least 7 days to habituate to the vivarium space prior to experimentation. Ai32 (Stock # 024109), Emx1Cre (# 005628), and vGluT2Cre (# 016963) breeders were originally obtained from the Jackson Laboratory. Conditional A1R knockout mice (A1Rflox) were a generous gift from Dr. Robert Greene (University of Texas Southwestern Medical Center). These mice express loxp sites flanking the major coding region for the A1R of exon 6. When introduced to cre recombinase via breeding with a cre-expressing mouse line, the A1R can be deleted from specific cell types. In depth information on A1Rflox mice can be found elsewhere^30^. Ai32/Emx1Cre, Ai32/vGluT2Cre, A1Rflox/Emx1Cre, and A1Rflox/vGluT2Cre mice were all bred and genotyped within our facility at the Indiana University School of Medicine. All mice were group housed and maintained on a standard 12-h light/dark cycle (lights ON at 0700). The temperature and humidity of the vivarium space were kept at 20 °C and 50%, respectively. Mice were between 60 and 120 days of age at the time of experimentation. All procedures described herein were approved by the Indiana University School of Medicine Animal Care and Use Committee and closely followed the guidelines for the care and use of laboratory animals as promulgated by the National Institutes of Health.

### Gene expression analysis

Our general quantitative protocol was previously described^31^. Briefly, mice were deeply anesthetized and decapitated. Cortical and thalamic tissue were rapidly dissected in an RNAse-free environment and samples were stored in RNAlater at – 80 °C until analysis. A1R mRNA expression in cortical and thalamic tissue from naïve adult male A1Flox/Emx1Cre+ and A1Flox/Emx1Cre-− mice as well as A1Flox/vGluT2Cre+ and A1Flox/vGluT2Cre− mice was measured via quantitative PCR. The probe for the A1R was obtained from Bio-Rad (Assay ID: qMmuCEP0056638; Hercules, CA, USA). Relative levels of A1R expression were determined via normalization to the endogenous control GAPDH and mRNA samples for each individual animal were run in triplicate as technical replicates.

### Brain slice preparation

Brain slices were collected for electrophysiology as previously described^31,32^. Mice were deeply anesthetized via isoflurane and euthanized via decapitation. Brains were rapidly excised and transferred to an ice-cold, oxygenated (95% O_2_/5% CO_2_ bubbled) cutting solution containing (mM): 30 NaCl, 4.5 KCl, 1 MgCl_2_, 26 NaHCO_3_, 1.2 NaH_2_PO_4_, 10 glucose, and 194 sucrose. 280 µm coronal brain slices containing the striatum were prepared on a VT1200S vibratome (Leica, Wetzlar, Germany). Sections were rapidly transferred to 32 °C, oxygenated artificial cerebrospinal fluid (aCSF) containing (mM): 124 NaCl, 4.5 KCl, 2 CaCl_2_, 1 MgCl_2_, 26 NaHCO_3_, 1.2 NaH_2_PO_4_ and 10 glucose for 1 h, after which they were held at room temperature until recordings were made (up to 8 h post-slicing).

### Electrophysiology

Our general slice electrophysiology protocol followed our previously described methodology^31,32^. Whole-cell voltage clamp recordings were made using a Multiclamp 700B amplifier and Digidata 1550B (Molecular Devices, San Jose, CA, USA). For recording, brain slices were moved to a recording chamber, held at 32 °C, and continuously perfused with oxygenated aCSF at a rate of ∼ 1.5 ml/min. To isolate excitatory transmission, picrotoxin (50 µM) was added to the aCSF in all experiments. Slices were visualized on an Olympus BX51WI microscope (Olympus Corporation of America, Center Valley, PA, USA). MSNs in the DMS and DLS were confirmed by their membrane resistance and capacitance.

For whole-cell recordings, borosilicate filamented glass recording pipettes (World Precision Instruments, Sarasota, FL, USA) registering 2–4 MΩ of resistance were filled with an internal solution (295–310 mOsm) containing (mM): 120 CsMeSO3, 5 NaCl, 10 TEA-Cl, 10 HEPES, 5 lidocaine bromide, 1.1 EGTA, 0.3 Na-GTP and 4 Mg-ATP. All recordings were filtered at 2.2 kHz and digitized at 10 kHz. Data were acquired using Clampex 10 software (Molecular Devices). Series resistance was continuously monitored and only cells with a stable access resistance (less than 25 MΩ and that did not change more than 15% from baseline average) were included for data analysis.

### A1R-SD

**Figure 1:**
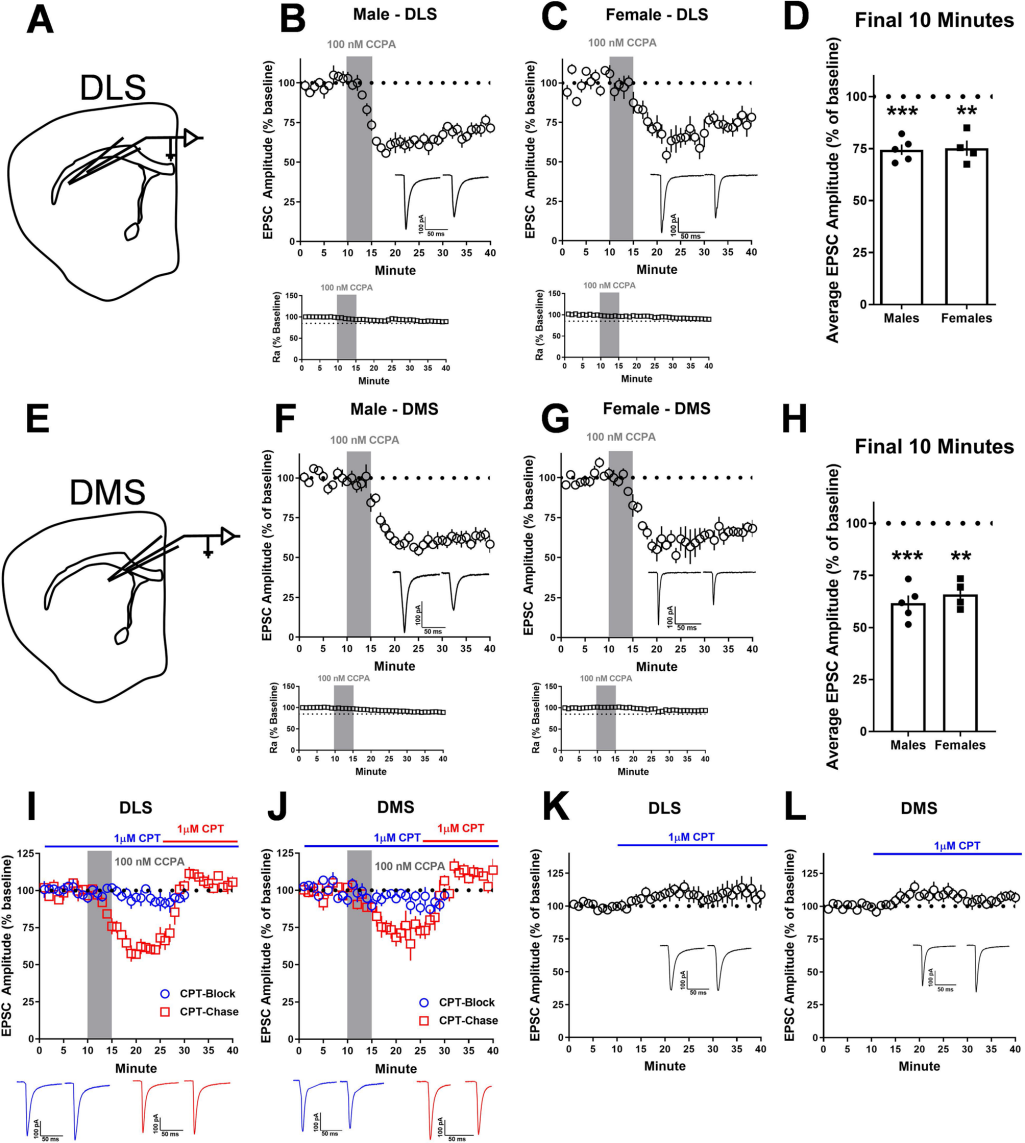
A1R-mediated synaptic depression occurs in both the dorsolateral (DLS) and dorsomedial (DMS) striatum of male and female C57/BL/6J mice. (**A**) Schematic of the recording location for DLS electrophysiological recordings. (**B**) Time course of CCPA-induced (100 nM for 5 min) A1R-SD in the DLS of male mice after a 10-min baseline of stable electrically evoked excitatory postsynaptic currents (EPSCs). (**C**) Time course of A1R-SD in the DLS of female mice. **D**) Average relative EPSC amplitude relative to baseline in the final 10 min of recording from male and female DLS. (**E**) Schematic of the recording location for DMS electrophysiological recordings. (**F**) Time course A1R-SD in the DMS of male mice. (**G**) Time course of A1R-SD in the DMS of female mice. (**H**) Average relative EPSC amplitude relative to baseline in the final 10 min of recording from male and female DMS. (**I**, **J**) In the CPT-Block experiments (data represented by blue circles), the A1R antagonist CPT (1 μM) was continuously present during a 30-min recording wherein CCPA was bath-applied for 5-min after a 10-min baseline, as usual. In the CPT-Chase experiments (data represented by red squares), A1R-SD was induced as usual and CPT was added to the bath beginning at min 25 of recording. Block and Chase experiments were only conducted in male mice. (**K**, **L**) Antagonist alone experiments wherein CPT was washed onto the slice after a stable 10-min baseline. Antagonist alone experiments were conducted in male and female mice, but data are pooled due to a lack of statistically significant sex differences. Data represent mean ± SEM. **p < 0.01, ***p < 0.001 vs. ‘100’. *n* = 4–14 cells and 2–6 animals per condition. For traces, EPSCs for the baseline period and final 10 min of recording were averaged from representative cells.

A similar pharmacologically-driven plasticity protocol was followed as previously described^31^. To generate EPSCs, a Teflon-coated tungsten bipolar stimulating electrode (PlasticsONE, Roanoke, VA, USA) was placed at the border of the corpus callosum and the DMS or DLS (Fig. 1A,E). Neurons were held at a − 60 mV holding potential and EPSCs were evoked every 20 s via a DS3 Isolated Current Stimulator (Digitimer, Ft. Lauderdale, FL, USA) and the intensity was adjusted until a stable response between − 200 and − 400 pA was observed. In optogenetic recordings in Ai32/Emx1Cre and Ai32/vGluT2 mice, optical EPSCs (oEPSCs) were evoked every 30 s via a 5 ms pulse of 470 nm blue light delivered by field illumination through the microscope objective. After a stable 10-min baseline, the A1R agonist 2-Chloro N6-cyclopentyladenosine (CCPA; 100 nM; Sigma Aldrich, St. Louis, MO) was briefly washed onto the slice for 5 min. Depending on the experiment, recording then continued for an additional 15–25 min post-CCPA. Ai32/Emx1Cre and Ai32/vGluT2 mice have been extensively used by various laboratories to study differential synaptic physiology at cortical versus thalamic inputs the striatum, respectively^33–35^.

To determine the receptor specificity of A1R-SD, antagonist experiments were also conducted. In the antagonist “block” experiments, EPSCs were electrically-evoked in DLS and DMS MSNs from male C57BL/6J slices while the A1R antagonist 8-cyclopentyl-1,3-dimethylxanthine (CPT; 1 µM; Tocris, Bristol, UK) was constantly in the bath and CCPA was washed onto the slice as usual. In antagonist “chase” experiments, A1R-SD was generated as usual, but CPT was introduced to the bath at the time point of peak CCPA response (minute 25). Finally, a separate experiment examined basal adenosine tone by washing CPT onto slices after a stable 10-min baseline.

### Miniature EPSCs

Measurements of miniature EPSCs (mEPSCs) were conducted to determine whether A1R-LTD occurred via a pre- and/or post-synaptic mechanism. In addition to picrotoxin, 500 nM tetrodotoxin was added to aCSF to isolate mEPSCs. After patching into MSNs and allowing 5–10 min for stabilization, a 3-min gap-free recording was taken as a baseline. CCPA was then washed onto the slice for 5-min and a second 3-min gap-free recording was taken 10 min later.

### Excitability

Cells were recorded in current clamp mode, allowing them to sit at their natural resting membrane potential. Increasing current steps (− 200pA to + 400pA in 50 pA increments) were injected for 500 ms every 10 s. The following excitability parameters were measured: resting membrane potential (RMP), input resistance, action potential (AP) threshold potential, AP peak, AP half‐width, and AP frequency. For the AP threshold potential, AP peak, and AP half‐width parameters, data from the first current step that produced APs were used. After an initial recording to establish baseline parameters, CCPA was washed onto the slice for 5 min as was done in the A1R-SD experiments and a second recording was taken 10 min later. No treatment recordings (no CCPA) from additional cells were also collected to control for repeated measurement effects. A K‐gluconate internal solution was used and contained (in mM) 4 KCl, 10 HEPES, 4 MgATP, 0.3 NaGTP, 10 phosphocreatine, 126 K‐gluconate. In addition to picrotoxin, the aCSF bath also contained NBQX (AMPA receptor antagonist; 5 μM) and AP-5 (NMDA receptor antagonist; 50 μM).

### Statistical analysis

With the exception of the initial A1R-SD experiment (Fig. 1A–H), all data and analyses are presented collapsed on sex because it was not a significant factor in any of the initial analyses. A1R-SD data were processed via pClamp 10.6 software (Molecular Devices, San Jose, CA, USA) and mEPSC data were processed using Mini-Analysis software (Synaptosoft Inc., Fort Lee, NJ, USA). Statistical analyses were conducted using GraphPad Prism 8 (GraphPad, San Diego, CA, USA). Data were analyzed via paired (mEPSC data) or unpaired (all other data) t-tests and the significance level was set at *p* < 0.05. Normality was evaluated via the Shapiro–Wilk test and non-normal data were analyzed via the Mann–Whitney U test. To determine the magnitude of peak synaptic depression or whether SD occurred, one-sample t-tests compared mean EPSC amplitudes at peak CCPA response (min 21–25 of recording) and during the final 10 min of recording to theoretical baseline mean, ‘100%’ (i.e. significantly lower than 100% indicates significant depression, whereas a value significantly higher would indicate potentiation). Data are represented at the mean ± S.E.M.

## Results

To confirm that we could measure A1R-SD in the dorsal striatum as others have observed^3,22^, recordings were made from MSNs in the DLS (Fig. 1A) and DMS (Fig. 1E) of naïve male and female C57BL/6J mice. CCPA application produced a robust, LTD of EPSCs that lasted for at least 25 min following the conclusion of CCPA application in both the DLS (males: 74.43% ± 2.516, *t*_4_ = 10.17, *p* < 0.001 vs. 100%, n = 5 cells from 3 mice, Fig. 1B,D; females: 75.2% ± 3.668, *t*_3_ = 6.762, *p* < 0.01 vs. 100%, n = 4 cells from 2 mice, Fig. 1C,D) and DMS (males: 61.77% ± 3.676, *t*_4_ = 10.4, *p* < 0.001 vs. 100%, n = 5 cells from 3 mice, Fig. 1F,H; females: 65.94% ± 3.332, *t*_3_ = 10.22, *p* < 0.01 vs. 100%, n = 4 cells from 2 mice, Fig. 1G,H). This effect was statistically similar in both male and female animals in both subregions (*p*’s > 0.05). These data demonstrate that we are able to measure A1R-LTD in both DLS and DMS.

When the A1R antagonist CPT was continuously present in the bath, A1R-LTD was completely blocked in both DLS (94.1% ± 3, *t*_4_ = 1.981, *p* > 0.05 vs. 100%, n = 5 cells from 3 mice, Fig. 1I) and DMS (92.5% ± 3.99, *t*_4_ = 1.879, *p* > 0.05 vs. 100%, n = 5 cells from 3 mice, Fig. 1J). A CPT chase also reversed A1R-LTD in both DLS (106.6% ± 3.01, *t*_4_ = 2.177, *p* > 0.05 vs. 100%, n = 5 cells from 3 mice, Fig. 1I) and DMS (109.7% ± 1.16, *t*_3_ = 8.377, *p* < 0.01 vs. 100%, n = 4 cells from 3 mice, Fig. 1J). This experiment assessed whether A1R-LTD was a static (insensitive to CPT) or labile (reversible by CPT) LTD, both of which have been observed by others in striatum^36^. CPT application on its own did not significantly influence evoked EPSC amplitude in either the DLS (109.9% ± 7.67, *t*_9_ = 1.292, *p* > 0.05 vs. 100%, n = 10 cells from 5 mice, Fig. 1K) or DMS (104.9% ± 3.228, *t*_13_ = 1.511, *p* > 0.05 vs. 100%, n = 14 cells from 6 mice, Fig. 1L), indicating a lack of substantial basal adenosine tone in brain slices containing these subregions.

**Figure 2:**
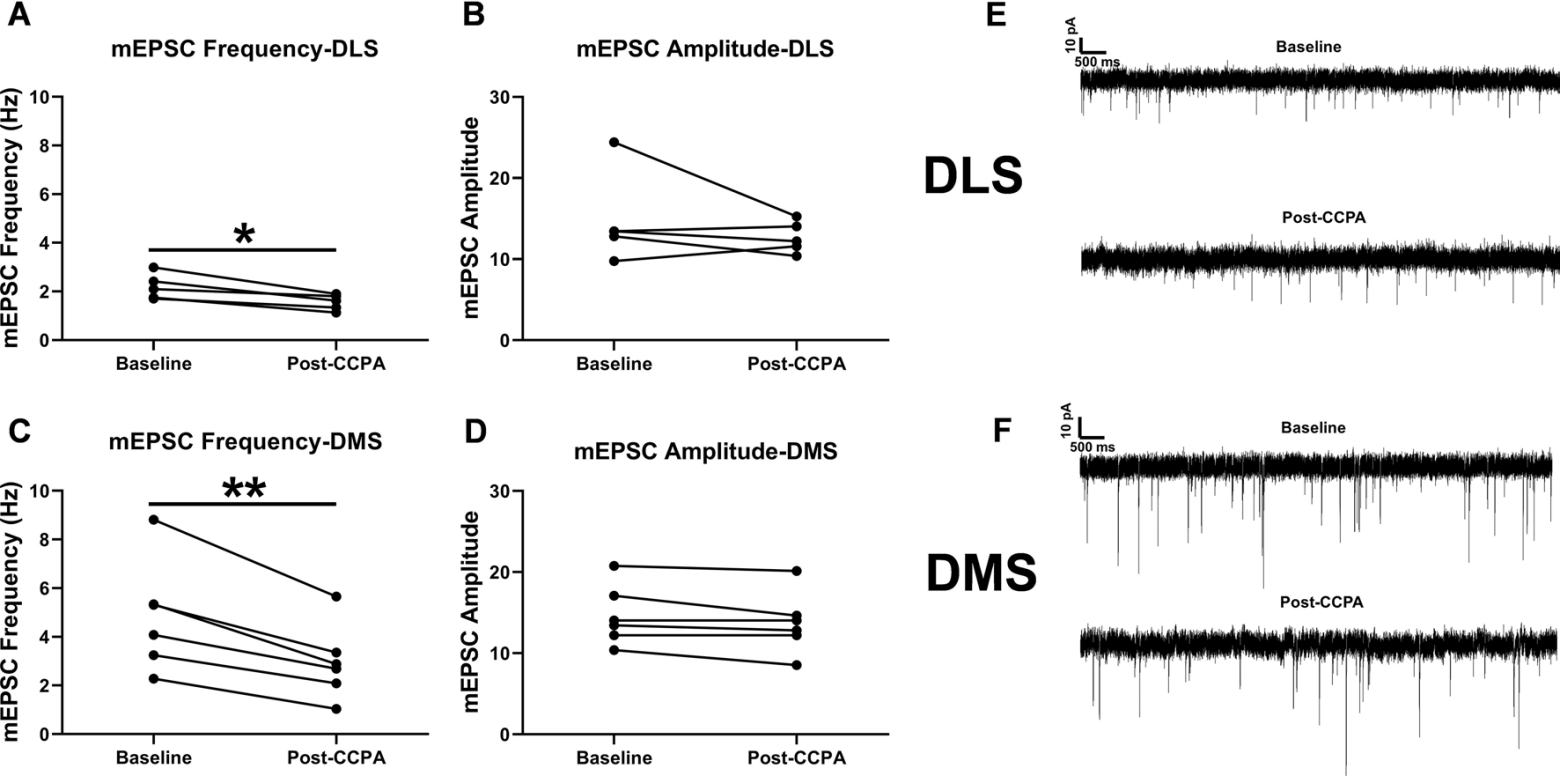
A1R synaptic depression occurs presynaptically in both dorsolateral (DLS) and dorsomedial (DMS) striatum. The (**A**, **C**) frequency and (**B**, **D**) amplitude of miniature EPSCs (mEPSCs) were quantified for 3-min recordings prior to and 10 min following a 5-min CCPA application in the DLS and DMS of naïve male C57BL/6J mice. (**E**, **F**) Representative traces of mEPSC recordings. Data represent mean ± SEM. *p < 0.05, **p < 0.05. *n* = 5–6 cells from 3 animals per region.

In mEPSC recordings, the frequency of events was significantly reduced compared to baseline in both DLS (*t*_4_ = 4.331, *p* < 0.05, n = 5 cells from 3 animals, Fig. 2A) and DMS (*t*_5_ = 5.843, *p* < 0.01, n = 6 cells from 3 animals, Fig. 2C) following CCPA application, suggesting that A1R activation reduced presynaptic glutamate release. mEPSC amplitude, however, was not altered by A1R activation in either subregion (*p*’s > 0.05, Fig. 2B,D). An additional excitability experiment was conducted to assess whether CCPA application influenced excitability of MSNs postsynaptically. Results indicated no effect of CCPA for any of the intrinsic membrane properties or excitability parameters in either DLS or DMS (Supplemental Figs. 1, 2). Collectively, these results indicate that dorsal striatal A1R-SD likely occurs through a presynaptic mechanism.

The dorsal striatal subregions receive anatomically and functionally distinct cortical and thalamic inputs. To begin to dissect the neural circuitry of dorsal striatal A1R-SD, electrophysiology recordings were made in slices from Ai32/Emx1Cre and Ai32/vGlutT2 mice. Ai32/Emx1Cre mice express the 470 nm light-sensitive cation channel channelrhodopsin2 (ChR2) in corticostriatal neurons^33^. Ai32/vGlutT2 mice express ChR2 in a number of regions, but the most robust expression is in the thalamus, the only photosensitive region in these mice that sends projections to dorsal striatum^33,34^. Thus, local blue light stimulation in dorsal striatum generates cortically- or thalamic-driven EPSCs in MSNs from Ai32/Emx1Cre and Ai32/vGlutT2 mice, respectively.

**Figure 3:**
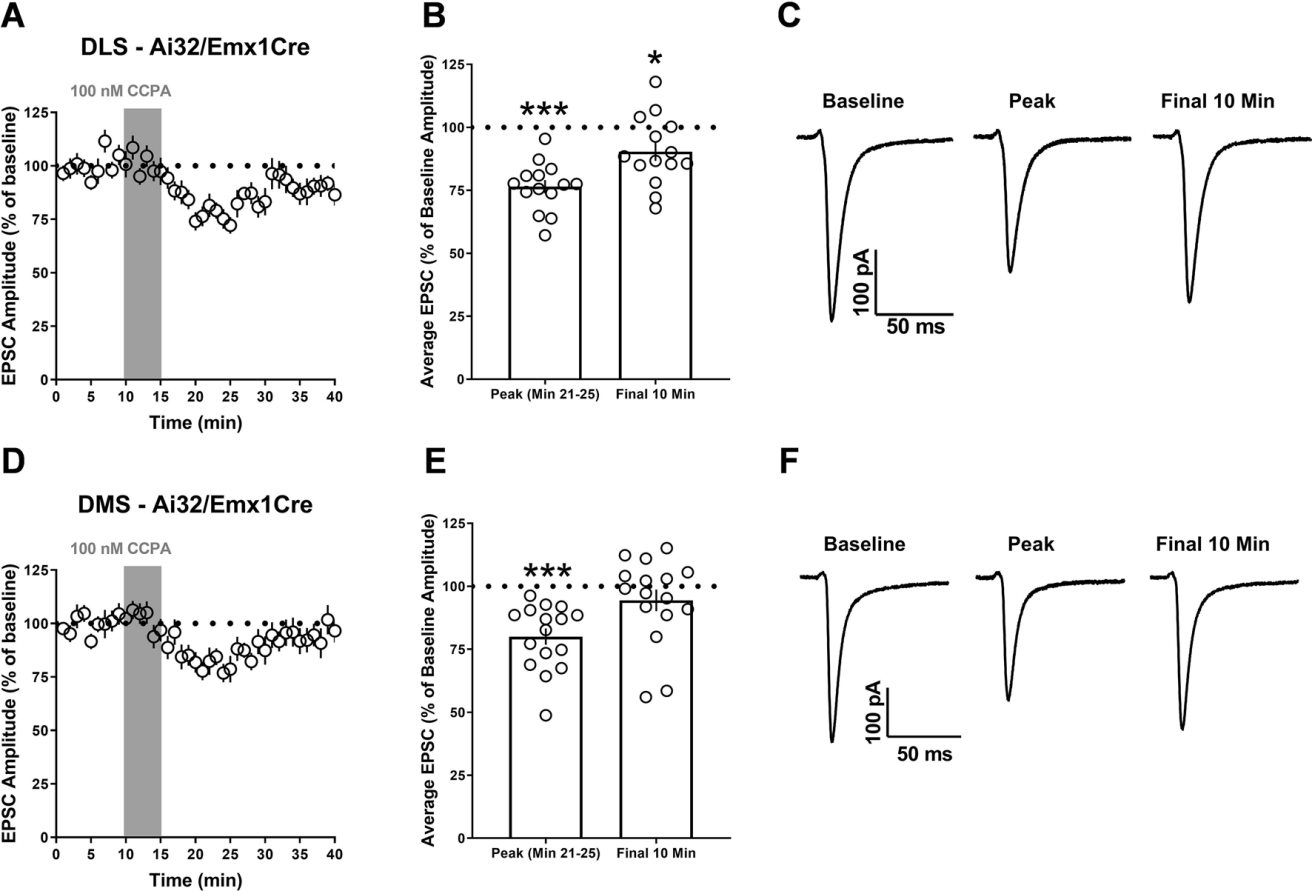
A1R synaptic depression occurs at Emx1-expressing inputs to the dorsolateral striatum (DLS), but not the dorsomedial striatum (DMS). (**A**, **D**) Time course of the response to CCPA in naive male and female (collapsed on sex) Ai32/Emx1Cre mice after a 10-min baseline of stable optically-evoked EPSCs. (**B**, **E**) Average EPSC amplitude for min 21–25 (peak CCPA response) and the final 10 min of recording relative to baseline. (**C**, **F**) Representative optically evoked EPSCs for baseline, peak, and final 10 min time points. Data represent mean ± SEM. *p < 0.05, ***p < 0.001 vs. ‘100’. *n* = 14–16 cells from 4 to 6 animals per sex.

**Figure 4:**
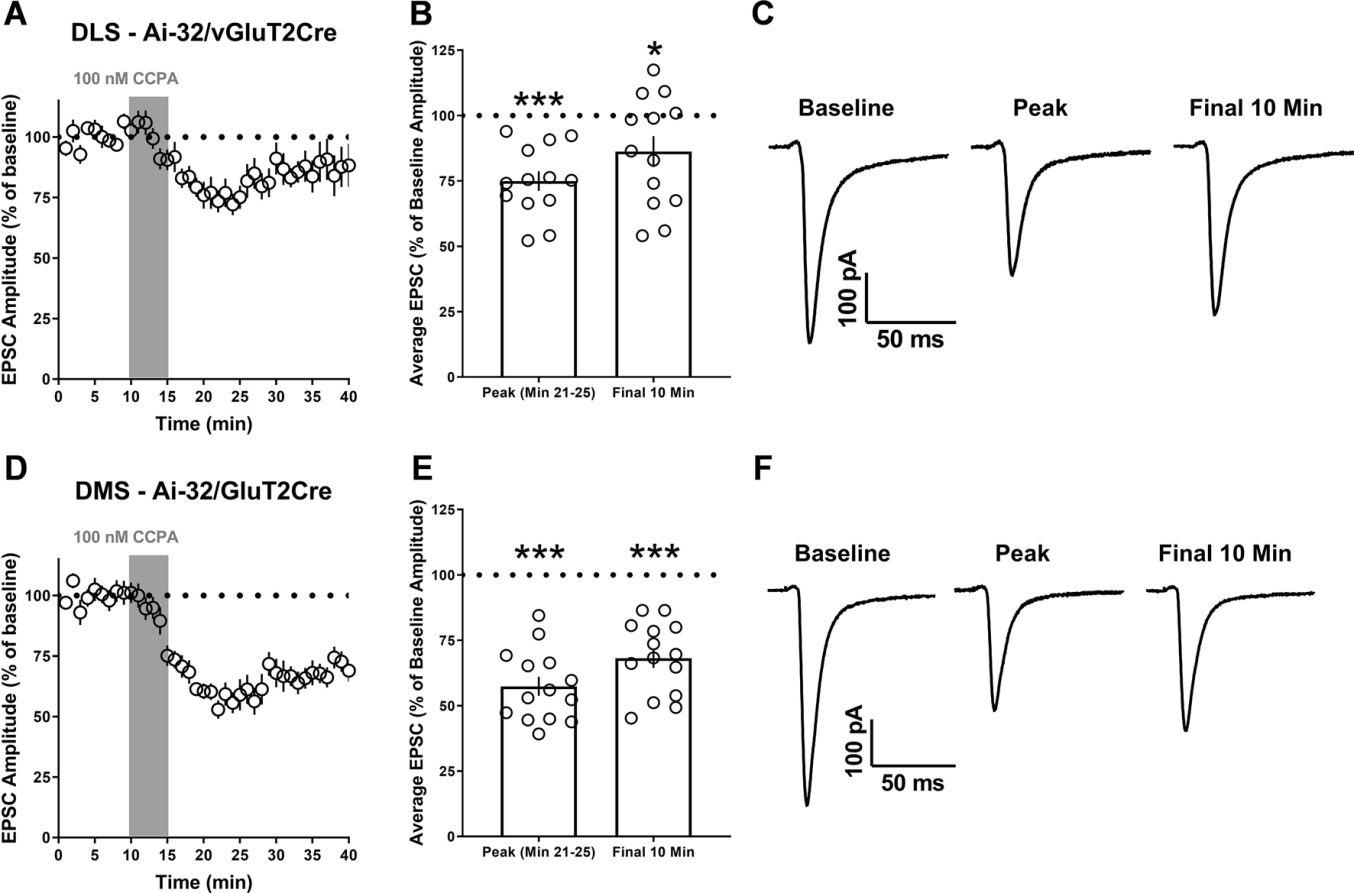
A1R synaptic depression occurs at vGluT2-expressing inputs to the dorsolateral (DLS) and dorsomedial (DMS) striatum. (**A**, **D**) Time course of the response to CCPA in naive male and female (collapsed on sex) Ai32/vGluT2Cre mice after a 10-min baseline of stable optically evoked EPSCs. (**B**, **E**) Average EPSC amplitude for min 21–25 (peak CCPA response) and the final 10 min of recording relative to baseline. (**C**, **F**) Representative optically evoked EPSCs for baseline, peak, and final 10 min time points. Data represent mean ± SEM. *p < 0.05, ***p < 0.001 vs. ‘100’. *n* = 13–14 cells from 4 to 6 animals per sex.

CCPA application produced a significant peak synaptic depression in the DLS MSNs of Ai32/Emx1Cre mice (76.41% ± 2.62, *t*_13_ = 9.011, *p* < 0.001 vs. 100%, n = 14 cells from 4–6 animals per sex, Fig. 3A–C) that decayed into a modest, but still present synaptic depression (90.33% ± 3.66, *t*_13_ = 2.641, *p* < 0.05 vs. 100%, Fig. 3A–C). Although significant peak synaptic depression was also observed in DMS MSNs (80.01% ± 3.23, *t*_15_ = 6.184, *p* < 0.001 vs. 100%, n = 15 cells from 4 to 6 animals per sex, Fig. 3D–F), significant depression was no longer evident in the final 10 min of recording (94.41% ± 4.3, *t*_15_ = 1.301, *p* > 0.05 vs. 100%, Fig. 3D–F). Similar to Ai32/Emx1Cre mice, CCPA produced significant peak synaptic depression (74.97% ± 3.72, *t*_12_ = 6.723, *p* < 0.001 vs. 100%, n = 13 cells from 4 to 6 animals per sex, Fig. 4A–C) and modest maintained synaptic depression in the DLS of Ai32/vGluT2Cre mice (86.24% ± 5.87, *t*_12_ = 2.343, *p* < 0.05 vs. 100%, Fig. 4A–C). In the DMS of these animals, however, CCPA produced a robust peak depression (57.4% ± 3.63, *t*_13_ = 11.72, *p* < 0.001 vs. 100%, n = 14 cells from 4 to 6 animals per sex, Fig. 4D–F) that remained as strong and significant synaptic depression in the final 10 min of recording (68.14% ± 3.7, *t*_13_ = 8.61, *p* < 0.001 vs. 100%, Fig. 4D–F).

**Figure 5:**
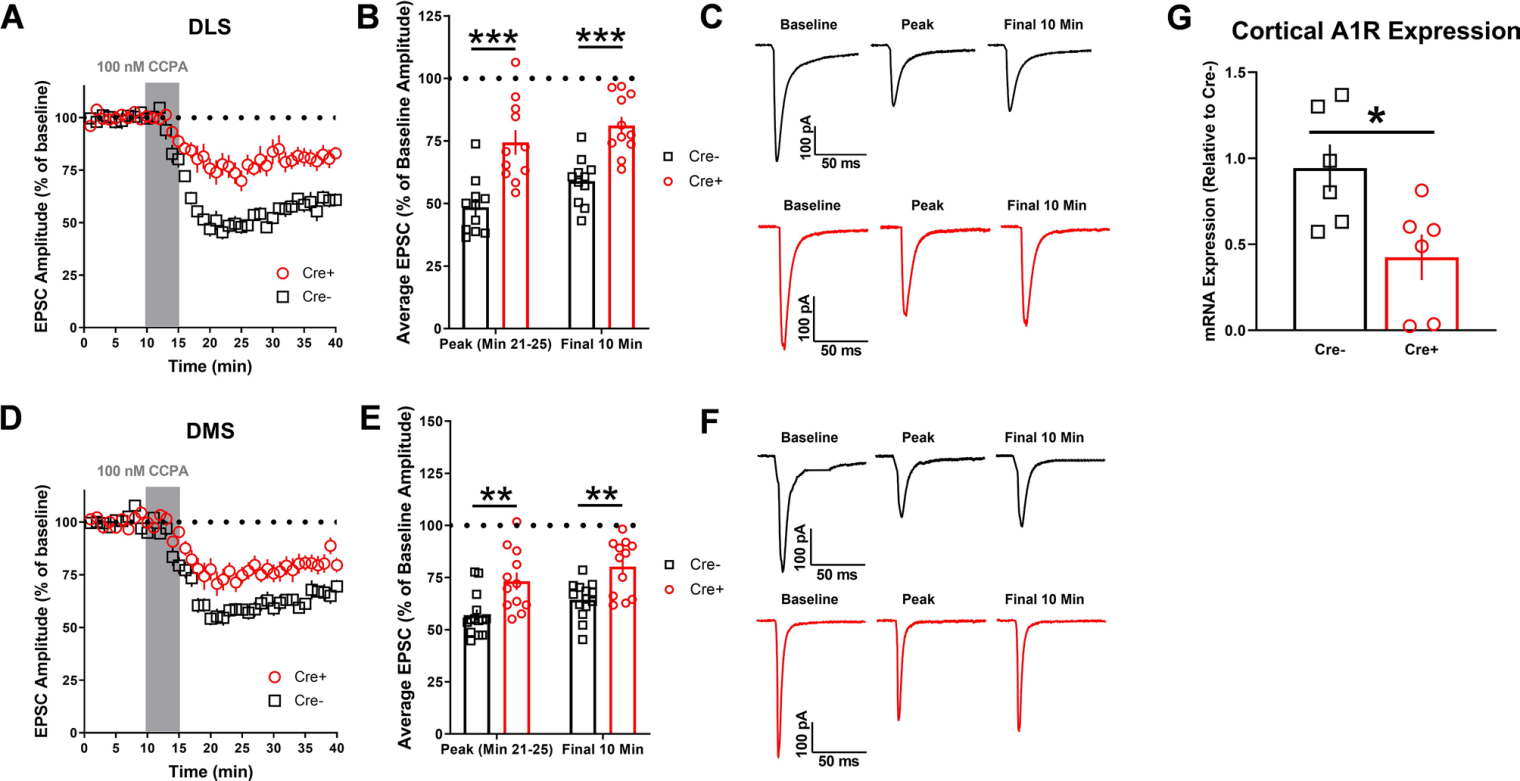
Focal knockout of A1Rs on Emx1-expressing inputs significantly interferes with A1R synaptic depression in both dorsolateral (DLS) and dorsomedial (DMS) striatum. (**A**,** D**) Time course of A1R-SD in male and female (collapsed on sex) A1Rflox/Emx1Cre+ and A1Rflox/Emx1Cre− mice after a 10-min baseline of stable electrically evoked EPSCs. (**B**, **E**) Average EPSC amplitude for min 21–25 (peak CCPA response) and the final 10 min of recording relative to baseline. (**C**, **F**) Representative electrically evoked EPSCs for baseline, peak, and final 10 min time points. (**G**) A1R expression in the cortex of male A1Rflox/Emx1Cre+ and A1Rflox/Emx1Cre− mice quantified by quantitative PCR. Data represent mean ± SEM. *p < 0.05, **p < 0.01, ***p < 0.001. For electrophysiology experiments, *n* = 11–14 cells from 4 to 6 animals per sex/genotype. For qPCR, *n* = 6 mice per genotype.

To further examine the neurocircuitry and the respective input contribution to dorsal striatal A1R-SD, we bred Emx1Cre and vGluT2Cre mice with A1Rflox conditional knockout mice. The resultant A1Rflox/Emx1Cre+ and A1Rflox/vGluT2Cre+ mice were generated to produce animals lacking A1Rs specifically at putative cortical or thalamic inputs to the dorsal striatum whereas Cre− littermates were intact. Using the electrical stimulation protocol as presented in Fig. 1, peak A1R-SD was significantly blunted in A1Rflox/Emx1Cre+ mice relative to their Cre− wildtype counterparts (DLS: *t*_20_ = 4.337, *p* < 0.001 Cre− vs. Cre+, n = 11 cells from 4 to 6 mice per sex; DMS: Mann–Whitney *U* = 25, *p* < 0.01 Cre− vs. Cre+, n = 14 cells from 4 to 6 mice per sex, Fig. 5A–F) and this effect persisted out to 25 min post-CCPA application (DLS: *t*_20_ = 4.909, *p* < 0.001 Cre− vs. Cre+; DMS: *t*_24_ = 3.662, *p* < 0.01 Cre− vs. Cre+, Fig. 5A–F). Analysis of cortical tissue from these mice via qPCR revealed a significant reduction in A1R expression (*t*_10_ = 2.723, *p* < 0.05, n = 6 mice per genotype; Fig. 5G).

**Figure 6:**
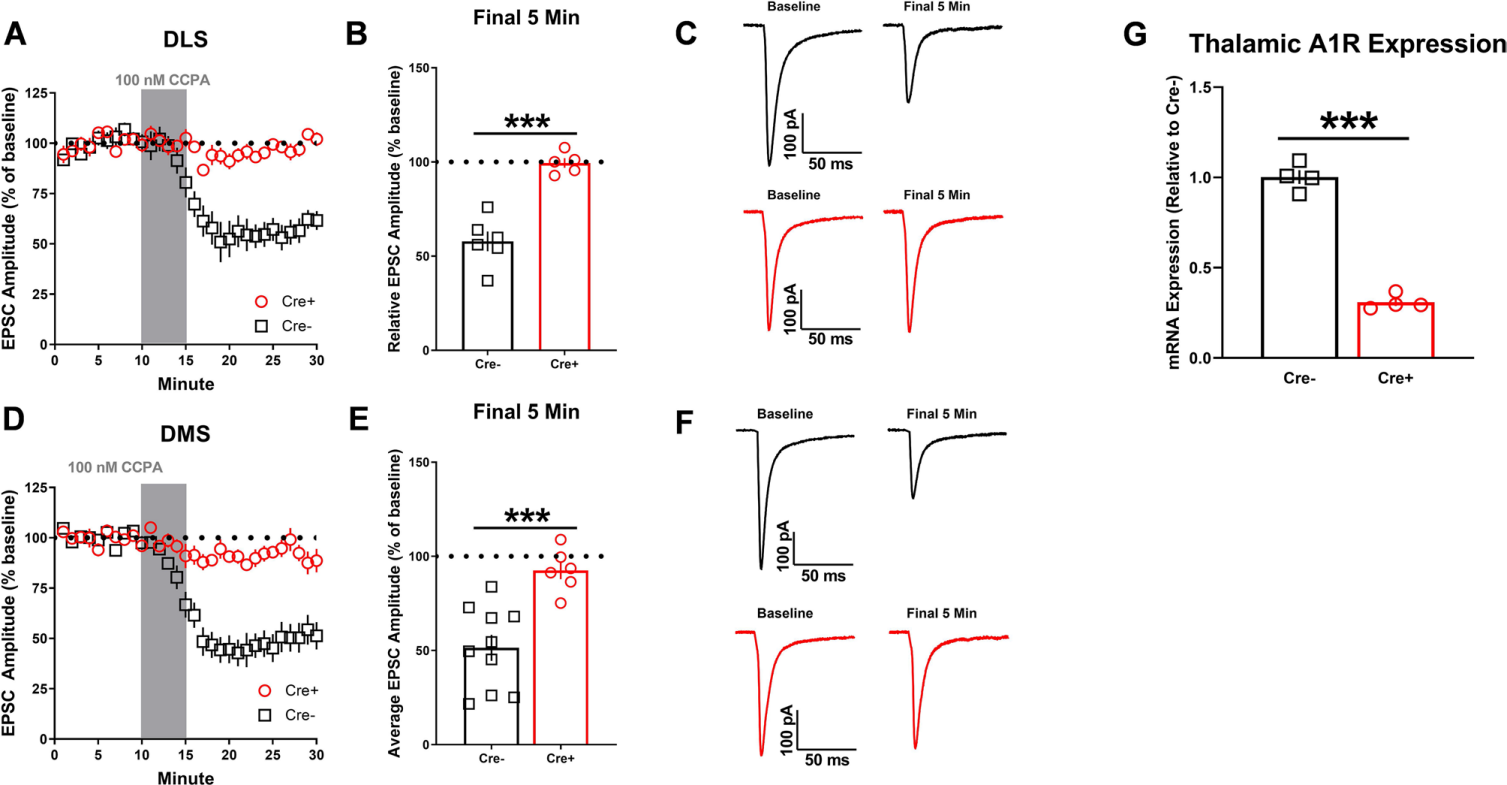
Focal knockout of A1Rs from vGluT2-expressing inputs ablates A1R synaptic depression in both dorsolateral (DLS) and dorsomedial (DMS) striatum. (**A**, **D**) Time course of A1R-LTD in male and female (collapsed on sex) A1Rflox/vGluT2Cre+ and A1Rflox/vGluT2Cre− mice after a 10-min baseline of stable electrically evoked EPSCs. (**B**, **E**) Average EPSC amplitude for final 5 min of recording (min 26–30) relative to baseline. (**C**, **F**) Representative electrically evoked EPSCs for baseline and final 5 min time points. (**G**) A1R expression in the thalamus of male A1Rflox/vGluT2Cre+ and A1Rflox/vGluT2Cre− mice quantified by quantitative PCR. Data represent mean ± SEM. ***p < 0.001. For electrophysiology experiments, *n* = 5–10 cells from 2–3 animals per sex/genotype. For qPCR, *n* = 4 mice per genotype.

For A1Rflox/vGluT2Cre + mice, A1R-SD was virtually absent in both the DLS and DMS relative to Cre- animals (DLS: *t*_9_ = 6.675, *p* < 0.001 Cre− vs. Cre+, n = 5–6 cells from 2–3 mice per sex; DMS: *t*_14_ = 4.228, *p* < 0.001 Cre− vs. Cre+, n = 6–10 cells from 2–3 animals per sex, Fig. 6A–F). Analysis of thalamic tissue via qPCR confirmed that the conditional A1R KO was effective (*t*_6_ = 16, *p* < 0.001, n = 4 mice per genotype; Fig. 6G).

## Discussion

This collection of experiments confirmed the existence of A1R-driven inhibitory plasticity of glutamate transmission (A1R-SD) within both subregions of the murine dorsal striatum. Results from optogenetic and conditional A1R knockout studies demonstrated that likely both cortical and thalamic inputs are involved in A1R-SD within the DLS, but there appears to be a strong bias for thalamic inputs to selectively express A1R-SD in the DMS. The ability of A1R activation to persistently inhibit glutamate release from these specific inputs likely influences the functional output of the dorsal striatal subregions. Thus, these findings may have significant implications for numerous perseverative neuropsychiatric disorders involving aberrations in dorsal striatal function that may ultimately promote inflexible and/or maladaptive behaviors.

This study significantly advances the striatal synaptic plasticity literature in a number of ways. To our knowledge, this is the first study to directly assess the A1R’s modulatory capacity at specific inputs to the dorsal striatum and completely novel transgenic mice (A1Rflox/Emx1Cre and A1Rflox/vGluT2Cre) were generated to make this assessment more thorough. In addition, this is the first study to carefully examine A1R-SD in dorsal striatal subregions, a distinction that has become increasingly critical due to the distinct inputs each receives and the behaviors each governs^8,9^. Lastly, most of the experiments presented here evaluated biological sex as a factor, a rarity in many published electrophysiology studies.

A1Rs have been known for some time to act as a regulators of long-term potentiation in the brain^37^, however their capacity to induce synaptic plasticity on their own has been understudied. Some investigators have identified A1R-SD in other brain regions including the hippocampus and cerebellum^36^. One recent study found A1R-SD using the same concentration of CCPA (100 nM) within the CA1 and CA2 regions of the hippocampus that likely occurred presynaptically, at a similar magnitude (60–75% of baseline EPSC magnitude), and persisted for a similar duration of time (40+ min)^38^. This similarity of A1R-SD observations in two highly distinct brain regions is noteworthy and interesting given the highly different inputs that each receives.

Earlier work in the striatum demonstrated that A1R activation can acutely inhibit excitatory transmission at both putative cortical and thalamic inputs^21^. In this previous study, an A1R agonist acutely decreased evoked EPSP magnitude measured in striatal MSNs that was produced by broad stimulation of putative cortical or thalamic fibers, although no time course or subregion specificity was examined. An older study by Lovinger and Choi^3^ may have provided the first observation of more persistent A1R-SD specifically in dorsal in striatum (lasting 50+ min), although the prolonged depressant effect of A1R agonist application in their experiment was likely misinterpreted as an inability to wash out drug effects. Our Ai32/Emx1Cre experiments in DMS (Fig. 3D–F), in relation to our other recordings, demonstrate that we are able to washout the effect of the A1R agonist, indicating that we are indeed measuring a form of depressive synaptic plasticity at other synapses.

More recently, putative A1R-SD was observed in dorsal striatal MSNs^22^. Our study expands greatly upon this prior work. Specifically, we demonstrate that an A1R antagonist blocks and reverses established A1R-SD, without affecting excitatory transmission itself (Fig. 1). We also found that a brief 5 min activation of A1Rs is sufficient to induce A1R-SD and does not require the more extended A1R agonist exposure (20 min) others used previously. Here we also show that A1R-SD also occurs in DMS in addition to the A1R-SD measured in the DLS in the previous work and further demonstrates that this form of SD likely occurs presynaptically (Fig. 2 and Supplemental Figs. 1, 2). We also demonstrate that A1R-SD occurs differentially at specific inputs to DLS and DMS (Figs. 3, 4, 5, 6). A1R-SD in DLS was only previously examined in direct pathway, D1 dopamine receptor-containing MSNs. Although we did not address indirect vs. direct pathway specificity, the consistency of our observations suggest that it likely occurs equally in both MSN types (i.e. bimodal distributions are not apparent in our data). Finally, we chose to examine A1R-SD in both male and female mice to assess sex effects, a variable that is rarely considered in dorsal striatal electrophysiology experiments. Although we found equivalent A1R-SD in male and female animals in all experiments, this consideration is a significant interpretational strength given the very different neurochemical environments of male and female mammals^39,40^.

SD can manifest in two different ways: static or labile^36^. As the name implies, static SD engages irreversible mechanisms and it is insensitive to the application of a receptor-specific antagonist. Labile SD, on the other hand can partially, or fully reverse in response to the application of an antagonist. As our ‘chase’ experiments indicate (Fig. 1I–J), A1R-SD is labile in both the DLS and DMS. These findings are in line with those by Trusel and colleagues (2015) who also observed a return to baseline values following antagonist application in their study. Various forms of both static and labile SD have been observed in the brain, including dorsal striatum^35,36^. The fact that A1R-SD is labile does not mitigate its relevance or importance, however. It is possible that application of the antagonist was able to change the conformational state of these A1Rs, causing the SD-induced conformation to change and reverse the effect on synaptic depression as has been observed with metabotropic glutamate receptor-mediated LTD^41^.

Alternatively, focal A1R activation by CCPA may sensitize the receptor to endogenous adenosine, thus amplifying its inhibitory signal. Although no evidence of significant adenosine tone was observed in either DLS or DMS (Fig. 1K–L), *this does not indicate the absence of endogenous adenosine*, but only that adenosine was not present in sufficient quantities to suppress presynaptic excitatory transmission in a basal state. Therefore, these basal adenosine levels may have effectively perpetuated A1R-SD following induction via CCPA application. This observation is consistent with a prior study also reporting a lack of effect of A1R antagonism on presynaptic glutamate transmission in dorsal striatum^22^. This previous study also demonstrated that A1R antagonism could block long-term depression induced by high frequency stimulation of presynaptic afferents in direct pathway D1 MSNs, suggesting that adenosine release within dorsal striatum may primarily occur as a consequence of high levels of presynaptic activity. Evidence for tonic inhibition by A1Rs has been found in other brain regions, however, such as the hippocampus. Application of the same A1R antagonist in the current study, CPT, produced increases in evoked EPSPs in the CA1 region of the of the hippocampus^42,43^. Interestingly, this effect became stronger over the course of postnatal development. Other work has also shown that the expression of A1Rs in hippocampal subregions and intracellular signaling influenced by A1R activation (e.g. phosphodiesterase and kinase activity) can shift during postnatal development^38^. Therefore, the effect of A1R activation on presynaptic function in the brain may be regionally- and developmentally-dependent as a function of both the level of receptor expression and the intracellular signaling cascades it influences.

Certain physiological conditions, such as alcohol intoxication^44^ and extreme sleepiness (Bjorness and Greene^1^) are also associated with augmented adenosine extracellular adenosine levels. Thus, there is noteworthy potential for the relevance of A1R-LTD for clinical conditions such as these. Nevertheless, future work will focus on deciphering the mechanism of this reversibility and clinical significance of A1R-SD.

In dorsal striatum electrophysiology experiments, electrical stimulation methods are indiscriminate and can generate EPSCs from a host of inputs. Given the distinct cortical and thalamic afferents the DLS and DMS each receive (Hunnicutt et al.^8^), the crucial next step was to determine the relative contribution of these inputs to A1R-SD in each subregion. Mild A1R-SD was observed in the DLS at both putative cortical (Ai32/Emx1Cre) and thalamic (Ai32/vGluT2) inputs (Figs. 3A–C and 4A–C). The magnitude of A1R-SD in both of these experiments (Ai32/Emx1Cre: 90.33% of baseline; Ai32/vGluT2Cre: 86.24% of baseline) did not reach the level achieved in the initial experiments using electrical stimulation (75% of baseline; Fig. 1D). We therefore propose that A1R-SD at cortical and thalamic inputs could be additive and collectively produced the result observed in the general electrical stimulation experiment. As for the DMS, we observed no A1R-SD at cortical inputs (Fig. 3D–F), but a robust A1R-SD at thalamic inputs (68.14%; Fig. 4D–F) that mirrored the effect observed via electrical stimulation (64% of baseline; Fig. 1H). These data suggest that there is a strong bias for thalamostriatal inputs governing A1R-SD in DMS.

To mechanistically confirm the role of A1Rs expressed presynaptically on these specific inputs to dorsal striatum in A1R-SD, we generated conditional knockout mice lacking A1Rs at putative cortical (A1Rflox/Emx1Cre+) and thalamic (A1Rflox/vGlut2Cre+) inputs. We observed a significant blunting of A1R-SD in both the DLS and DMS of A1Rflox/Emx1Cre+ mice, although it was not completely ablated. In A1Rflox/vGlut2Cre+ mice, both the acute and more protracted components of A1R-SD were strikingly absent in both the DLS as and DMS. Collectively, these data also support the notion that cortically- and thalamic-driven A1R-SD in DLS can be additive. In addition, it is also clear that A1R-SD is likely primarily governed by thalamostriatal inputs to DMS.

Although it is currently unclear, there are many potential mechanisms for how A1R-SD may occur within presynaptic neurons. A1Rs are G_i/o_ coupled receptors and their activation can reduce neurotransmitter release likelihood via inhibition of voltage gated Ca2 + channels, interfering with synaptic vesicle docking machinery, or the direct inhibition of adenylyl cyclase to halt cAMP production which ultimately inhibits the PKA signaling cascade^36^. A recent study by Caruana and Dudek demonstrated subregion specificity for A1R-SD mechanisms with the hippocampus^38^. Inhibition of NMDARs accentuated A1R-SD only within CA2 and inhibition of phosphodiesterase activity prevents the induction of A1R-SD only within CA1. This suggests that A1R-SD does not occur by any singular mechanism and that A1R-SD may occur via different intracellular signaling pathways in specific subregions of the brain. Future work will begin to elucidate the molecular mechanisms of presynaptic dorsal striatal A1R-SD within the DLS and DMS subregions.

As a general process, SD plasticity may help finetune the strength of various inputs to their target regions and thus ultimately influence neurocircuit dynamics^36^, disruptions of which may reflect pathologically excessive input from key projection regions that drive maladaptive behaviors. A1Rs are associated with these disorders which substantially impact patients’ quality of life, including substance use^45–47^, movement^6^, and sleep disorders^1,7^. It is conceivable that disrupted dorsal striatal A1R-SD may be an intriguing neuroadaptive process associated these various disease states. Given the unique roles of the DLS and DMS in cognitive-behavioral flexibility and the discrete anatomy of their inputs^8,9^, this newly characterized striatal plasticity offers many new exciting research opportunities.

## Supplementary Information

Supplementary Information
