# Supplementary material for: Input-selective adenosine A1 receptor-mediated synaptic depression of excitatory transmission in dorsal striatum
Source: Sci Rep. 2021 Mar 18;11:6345. doi: 10.1038/s41598-021-85513-x (PMC7973535; doi:10.1038/s41598-021-85513-x)
Supplement: Supplementary file 1 — Supplementary Information [file 41598_2021_85513_MOESM1_ESM.docx]

SUPPLEMENTARY MATERIAL

Input-Selective Adenosine A_1_ Receptor-Mediated Synaptic Depression of Excitatory Transmission in Dorsal Striatum

Brandon M. Fritz, Fuqin Yin, & Brady K. Atwood

**Figure S1.** CCPA application does not influence MSN intrinsic membrane properties or excitability in the DLS. Measures of **A**) resting membrane potential, (**B**) input resistance, (**C**) action potential threshold, (**D**) action potential peak amplitude, (**E**) action potential half-width, and (**F, G**) frequency of action potentials before and after CCPA application in Test cells and in the absence of CCPA in Control cells. Data represent mean ± SEM. **p*< 0.05 for main effect of repeated testing. *n =*9-10 cells from 4 animals each per condition.

**Figure S2.** CCPA application does not influence MSN intrinsic membrane properties or excitability in the DMS. Measures of **A**) resting membrane potential, (**B**) input resistance, (**C**) action potential threshold, (**D**) action potential peak amplitude, (**E**) action potential half-width, and (**F, G**) frequency of action potentials before and after CCPA application in Test cells and in the absence of CCPA in Control cells. Data represent mean ± SEM. **p*< 0.05 for main effect of repeated testing. *n =*8-10 cells from 4 animals each per condition.

**Supplemental Figure 1**

**
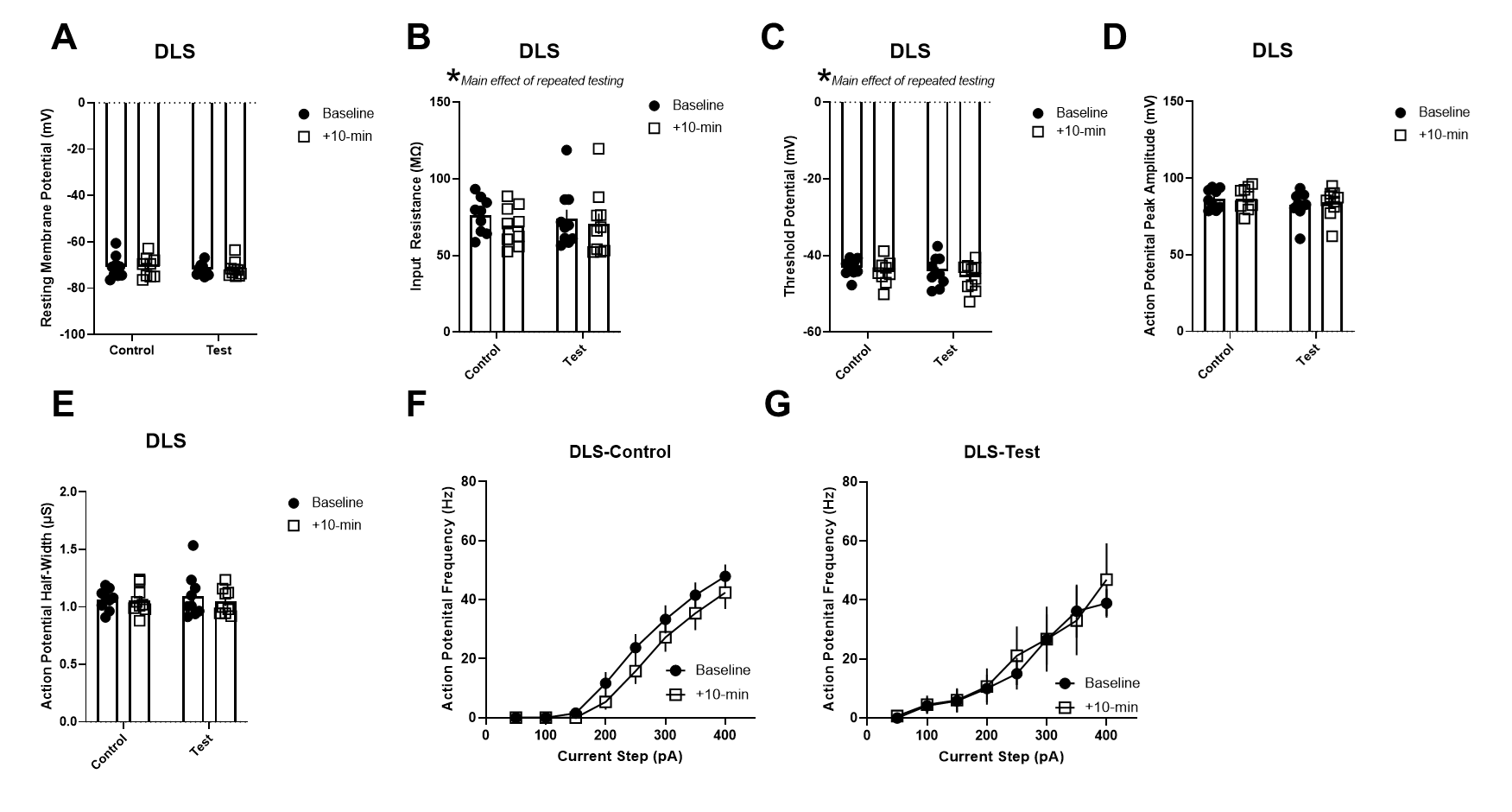
**

**Supplemental Figure 2**

**
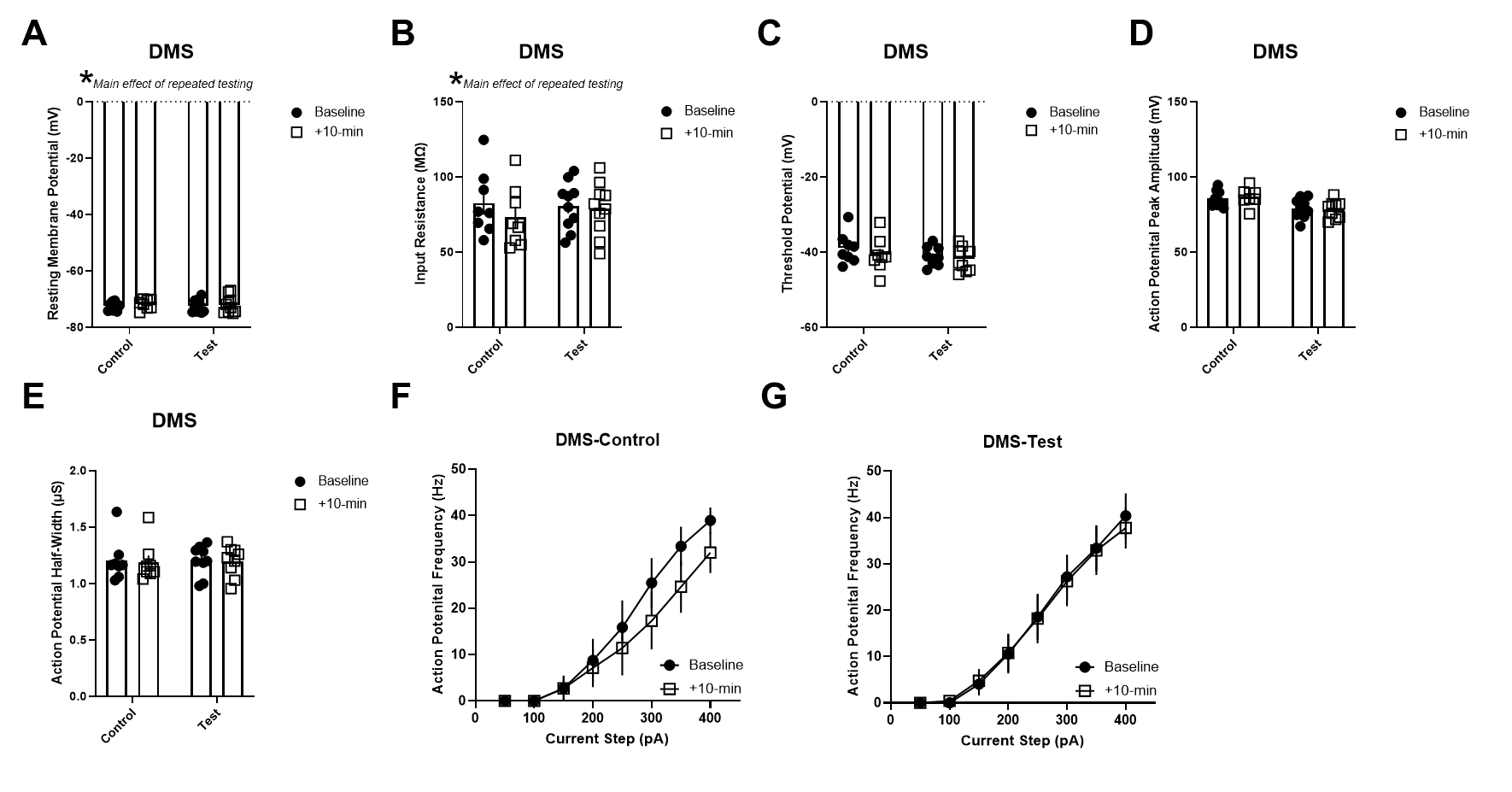
**
